# Radiological Detection of *Dracunculus medinensis*

**DOI:** 10.4269/ajtmh.17-0944

**Published:** 2018-05

**Authors:** Cristina Carranza-Rodríguez,, José Luis Pérez-Arellano

**Affiliations:** 1 Unidad de Enfermedades Infecciosas y Medicina Tropical, Complejo Hospitalario Materno Infantil de Gran Canaria, Las Palmas de Gran Canaria, Spain;; 2 Department of Medicine, Unidad de Enfermedades Infecciosas y Medicina Tropical, Complejo Hospitalario Materno Infantil de Gran Canaria, Las Palmas de Gran Canaria, Spain

**Figure 1.**
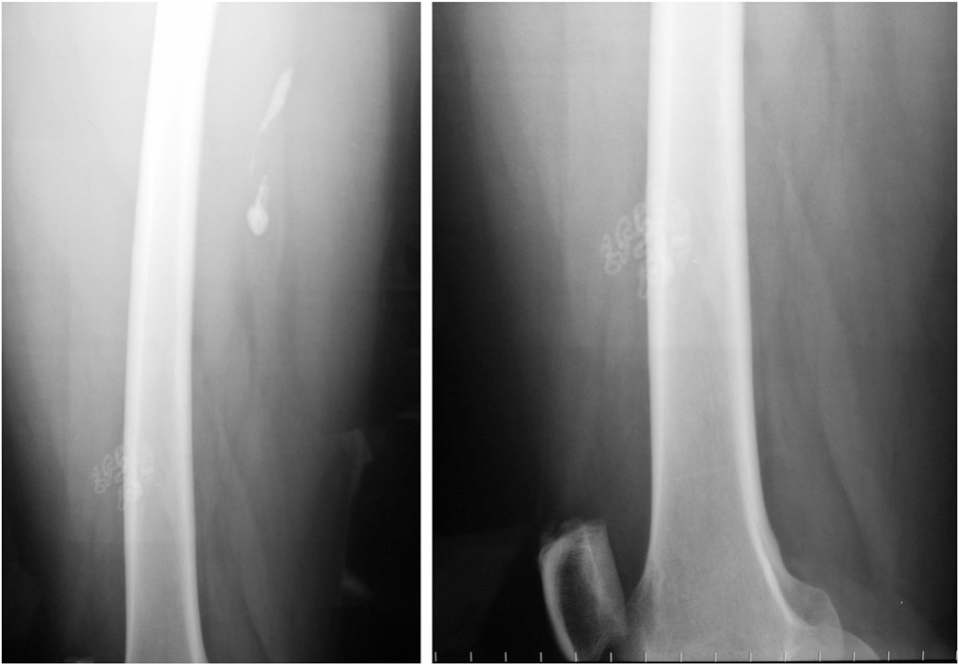
Guinea worm calcifications in a patient from Mali.

A 44-year-old female immigrant from Mali was referred to the Tropical Diseases Unit with a 1-year history of pain in the left thigh. The patient arrived in Spain 15 years ago and has never returned to her country. As part of her workup, radiography was performed, which revealed a calcified lesion, serpentine in appearance, in the distal aspect of the left thigh (Figure 1). This characteristic appearance led to the diagnosis of an old *Dracunculus medinensis* infection, which was unrelated to the patient’s presenting complaint (osteoarthritis) and did not require treatment.

Dracunculiasis is caused by a parasitic nematode known as *D. medinensis*, which is transmitted to humans by drinking contaminated water containing infected copepods. Once dead, the adult worm may undergo calcification, the typical location being in the lower extremities and usually with a long, string-like, serpiginous appearance.^1^ The radiologic differential diagnosis should include other parasitic diseases. Several filarial worms, specifically *Loa loa* and *Onchocerca volvulus*, may calcify, but these are much smaller and almost always seen in the hands and feet. In cysticercosis, patients have multiple “rice grain” calcifications that are oriented along the direction of the muscle fibers and are quite easy to identify and distinguish from *D. medinensis*. Only if the worm has partially disintegrated, the calcifications may appear more amorphous and causes problems in diagnosis. Our patient had the classic type of calcification (curvilinear calcification) seen in guinea worms in the extremities.

The World Health Organization estimated that there were approximately 3.2 million people infected in Africa in 1986. However, in 2016, only 25 cases were recorded in three countries: Chad, Ethiopia, and South Sudan.^2^ By 2020, dracunculiasis could be eradicated, not by pharmacological treatment or preventive vaccine, but by promoting community health education and behavior change.^3^
